# Coupled Transport Effects in Solid Oxide Fuel Cell Modeling

**DOI:** 10.3390/e24020224

**Published:** 2022-01-31

**Authors:** Aydan Gedik, Nico Lubos, Stephan Kabelac

**Affiliations:** Institute of Thermodynamics, Leibniz University Hannover, Welfengarten 1, D-30167 Hannover, Germany; kabelac@ift.uni-hannover.de

**Keywords:** solid oxide fuel cell (SOFC), non-equilibrium thermodynamics (NET), Dusty-Gas model (DGM), thermal diffusion, Soret effect, entropy production, exergy efficiency

## Abstract

With its outstanding performance characteristics, the SOFC represents a promising technology for integration into the current energy supply system. For cell development and optimization, a reliable quantitative description of the transport mechanisms and the resulting losses are relevant. The local transport processes are calculated by a 1D model based on the non-equilibrium thermodynamics (NET). The focus of this study is the mass transport in the gas diffusion layers (GDL), which was described as simplified by Fick’s law in a previously developed model. This is first replaced by the Dusty-Gas model (DGM) and then by the thermal diffusion (Soret effect) approach. The validation of the model was performed by measuring U,j-characteristics resulting in a maximum deviation of experimental to simulated cell voltage to up to 0.93%. It is shown that, under the prevailing temperature, gradients the Soret effect can be neglected, but the extension to the DGM has to be considered. The temperature and heat flow curves illustrate the relevance of the Peltier effects. At T=1123.15 K and j=8000 A/m^2^, 64.44% of the total losses occur in the electrolyte. The exergetic efficiency for this operating point is 0.42. Since lower entropy production rates can be assumed in the GDL, the primary need is to investigate alternative electrolyte materials.

## 1. Introduction

The demand for electrical energy has been steadily increasing in recent years, and, along with it, the importance of environmentally friendly energy converters. Electrochemical systems, such as fuel cells, are such alternative technologies for directly converting the chemical internal energy of a fuel into electrical energy. Hydrogen as an energy carrier becomes inexhaustible due to its simple production using solar-based carbon free energy sources and enables its use without direct CO_2_ emissions, making it environmentally friendly, also with regard to the Paris Climate Agreement.

The solid oxide fuel cell (SOFC) is a promising technology due to its superior performance indicators. These include the integration into the current energy supply system through the use of carbon-containing fuels, such as natural gas, the variability of the purity of the hydrogen required, and high efficiency due to the high operating temperatures. According to the current state of development, the electrical efficiency of SOFCs reaches values of up to 60–65% [1]. For the development of improved SOFCs, it is of great importance to be able to simulate the processes and the operating behavior taking place inside of the cells with the help of models. This is especially true as an experimental assessment of the ongoing processes inside the cell is prohibitive because of the temperature level around 1000 K. Many models already exist in the literature. These range from modeling individual layers within one cell to modeling the SOFC as a stack system. Three-dimensional models were set up in the work of Anderson et al. [2], Mauro et al. [3], Yakabe et al. [4], and Peksen et al. [5], in which the SOFCs are modeled based on the 3D finite element method. The momentum transport is described in these works with the help of the Navier–Stokes equations. In the work of Peksen et al., the SOFC is modeled, including peripheral components, such as the heat exchangers and the recirculation loops. Individual layers were investigated, for example, by Xu and Dang [6], Niu et al. [7], and Joos [8]. They model the porous electrodes of the SOFC in terms of their exact microstructure and investigate the relationship between the microstructure and the electrochemical reactions at the three-phase boundaries. In all these models, the transport processes are described using the classical empirical equations for the transport of heat, charges, molecules, and momentum. Why these empirical equations may not be sufficient for an exact description of the transport mechanisms in an SOFC is addressed in this work.

The performance and lifetime in an SOFC are determined by large temporal and spatial temperature gradients. Zeng et al. [9] shows an extensive literature review on heat transfer in SOFC stacks and derived thermal management methods. The study shows that excessive temperature gradients in SOFCs can lead to delamination and cracks in the electrolyte and electrodes. Depending on the operating point, temperature gradients in a planar co-flow SOFC along the electrolyte in the main flow stream of up to 30 K/cm can be reached. To avoid damage in the SOFC, a temperature gradient of 10 K/cm should not be exceeded [10]. Such temperature gradients can be reduced in the system by means of an effective heat management.

Temperature gradients are also caused by local heat generation and by flow field arrangement. Two types of heat generation are relevant in an SOFC. Reversible heat describes the reaction entropy of the system as a function of temperature, while irreversible heat is caused by dissipated energy. Dissipated energy arises due to ohmic resistance and overvoltages at the electrodes. The heat generation due to an electric current is described by means of the first Joule’s law, which is why the irreversible heat is also called Joule’s heat. In contrast, the electrochemical reaction at the three-phase interface leads to a thermoelectric effect in which reversible heat is released or absorbed at each electrode, also called Peltier heat.

In addition to a temperature gradient or a gradient in the electric potential, a gradient in the chemical potential of a species can also lead to a heat flow, called the Dufour heat. The influence of this coupling of multiple transport mechanisms caused by gradients of different intensive variables on the heat flux in a PEMFC is investigated in Reference [11], where individual effects on heat generation (Fourier, Peltier, and Dufour) are examined. As expected, at typical current densities, there is a net heat flux pointing out of the anode and cathode. An increasing current density leads to a higher potential difference and a higher electro-osmotic effect, which increases the Peltier and the Dufour effect. However, above a current density of $9700\;{A/m}^{2}$, Fourier heat dominates due to the temperature rise of the cathode surface caused by the irreversible activation overvoltage. Valadez Huerta [12] investigates, among other things, the contribution of the Peltier effect to the heat fluxes in an SOFC. A significant contribution is shown to come from the electrolyte, where the heat flux can flow from a lower to a higher temperature within the electrolyte due to the coupling effects mentioned above.

At the same time, the temperature gradients which develop affect the charge flow (Seebeck effect) and the material flow (Soret effect). The Seebeck effect describes a charge transport caused by a temperature gradient. Thus, due to this thermoelectric effect, a voltage can be measured between two different conductors when a temperature gradient is imposed on them. The magnitude of this voltage is described by the Seebeck coefficient, which depends on the material and the temperature. In the works of Kjelstrup et al. [13,14], a temperature gradient is applied to an electrochemical cell in order to measure the Seebeck coefficient. From this, the entropy of the oxygen ions transported in the electrolyte can be determined, which can also be used to calculate the Peltier coefficient.

Fick’s law, the Stefan–Maxwell diffusion, or the Dusty-Gas model (DGM) are widely used to describe mass transport within a cell. In Reference [12], the coupling of the mass flow with other flows is neglected. Using Fick’s law, the concentrations of the individual components are calculated using multi-component diffusion coefficients from the work of Costamagna et al. [15]. In the work of Suwanwarangkul et al., the approaches for describing the mass transport by means of Fick’s law, Stefan–Maxwell diffusion, and the DGM for determining the concentration overvoltage at the anode are examined. The modeling with the DGM achieves the best results, whereas the approaches using Fick’s law and Stefan–Maxwell diffusion are good approximations, especially for small electrical current densities and large pore diameters [16].

In Reference [11], the modeling of a PEMFC using non-equilibrium thermodynamics (NET) takes into account the Soret effect, whereby a material flow is driven by a temperature gradient. In operating ranges of high electric current densities, a significant influence of the Soret effect is observed in the membrane of the PEMFC. This is due to the strongly increasing temperature gradients in the membrane with increasing electric current density.

Considering these results, the coupling effects for a reliable description of the transport mechanisms in fuel cells cannot be neglected. It is not sufficient to address these coupling effects individually and simply add them up. It is very important to take an integrated approach as will be shown below. An integrated coupling of several transport processes can be based on theory of non-equilibrium thermodynamics (NET), which has only been considered in a few models so far. The first known model based on NET is the 1D model by Kjelstrup and Bedeaux [17], which can be used to calculate the potential field and the temperature curve of the cell. Within the NET theory, a generalized flux $J_{i}$ results from the linear combination of all occurring forces $X_{j}$ [18]:

(1) $$J_{i}=\sum_{j=1}^{N}L_{\mathrm{ij}}X_{j},$$

where the phenomenological coefficients $L_{\mathrm{ij}}$, also called conductances, are the connection between forces and fluxes. Forces are gradients of intensive variables. From the phenomenological equations, a matrix with the $N^{2}$ conductances $L_{\mathrm{ij}}$ necessary for modeling is obtained. By Onsager’s reciprocity relation $L_{\mathrm{ij}}=L_{\mathrm{ji}}$, the problem reduces to the upper and lower triangular matrices of this $L_{\mathrm{ij}}$ matrix, including the coefficients $L_{\mathrm{ii}}$ of the main diagonal [19].

For the optimization of a technical system, it is of great importance to understand these local transport mechanisms and their driving forces, as they all dissipate energy, i.e., create entropy. With the help of the local entropy production rate, loss mechanisms can be identified and quantified as exergy losses. In terms of NET, the local volume specific entropy production rate ${\dot{\sigma }}_{i}$ due to the transport process is generally calculated by multiplying a flux $J_{i}$ by the respective corresponding force $X_{i}$ [20]:

(2) $${\dot{\sigma }}_{i}=J_{i}\cdot X_{i},$$

thus allowing the second law of thermodynamics to be integrated into the model approach. However, if several fluxes are present simultaneously in a process, the total local entropy production is calculated by summing all products of occurring fluxes $J_{i}$ with the respective corresponding force $X_{i}$ [20]:

(3) $$\dot{\sigma }=\sum_{i}J_{i}X_{i}\geq 0.$$

Sauermoser et al. concluded that, in a PEMFC, the total local entropy production rate is highest at the cathode, which is explained by the potential profile. In contrast, Valadez et al. break down the total local entropy production rate of an SOFC into its individual contributions. In the gas diffusion layers (GDL), the diffusion process provides the largest contribution to the local entropy production rate, with losses at the cathode GDL being higher than losses at the anode GDL. In the electrolyte, the local entropy production rate is dominated by ionic conduction. If the Peltier effect is neglected, the GDLs show reduced values for the heat flux and potential contribution. As a result, a change of direction of the heat flux in the electrolyte happens. In both cases, the highest local entropy production rate results in the electrolyte, with up to 73%.

Our work aims at providing a consistent model approach for a single solid oxide fuel cell based on the NET theory. The 1D NET model by Valadez Huerta [12] provides the starting model for our approach. However, the mass transport described by Fick’s law is extended by transport equations that consider the coupling of several transport mechanisms. In addition, the phenomenological equations of the electrolyte are transformed to model the electrolyte with measurable coefficients. With the localization of the individual loss mechanisms, the system is then evaluated exegetically.

## 2. Materials and Methods

### 2.1. The Thermodynamic System—Solid Oxide Fuel Cell (SOFC)

**Figure 1:**
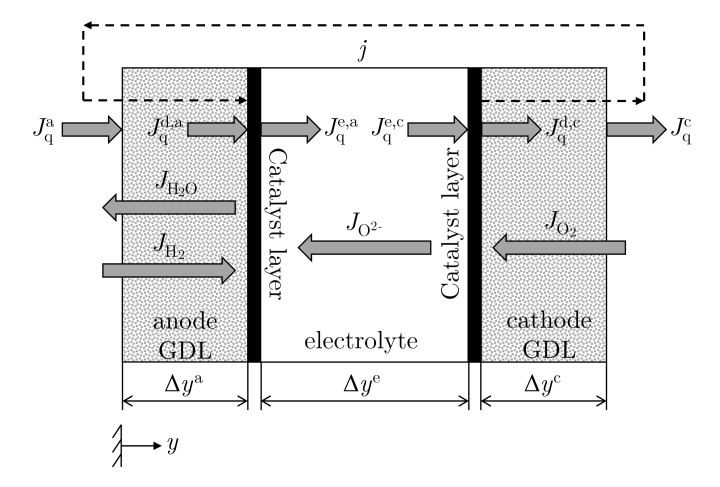
Model of the solid oxide fuel cell (SOFC), including the electric current density *j*, all heat fluxes $J_{q}$, and molar fluxes $J_{i}$.

For modeling purposes, the SOFC is divided into five different layers; see Figure 1. The membrane electrode assembly comprises a homogeneous bulk phase, which is separated by two surfaces. The boundaries to these two surfaces are two GDLs. Via the flow field plates, which are not considered in this model, the reactants are homogeneously distributed to the anode and cathode GDL. The surfaces also form interfaces between the three homogeneous bulk phases. The three homogeneous bulk phases, i.e., electrolyte membrane (e), anode GDL (a), and cathode GDL (c), each have a thickness of $\Delta y^{i}$.

The oxygen reduction reaction (ORR) takes place on the surface of the cathode catalyst layer, and, at the surface of the anode catalyst layer, the negative oxygen ions react, together, with hydrogen in the hydrogen oxidation reaction (HOR):

(4) $$\begin{array}{c} 1/2\;O_{2}\;\left(g\right)+2\;e^{-}⇄O^{2-}, \end{array}$$

(5) $$\begin{array}{c} H_{2}\;\left(g\right)+O^{2-}⇄2\;e^{-}+H_{2}O\;\left(g\right). \end{array}$$

Atomic vacancies in the electrolyte allow diffusion of the negative oxygen ions. In this study, 8 mol% yttria-stabilized zirconia (8YSZ) is investigated as the electrolyte material. The electron transport is ensured via wires that are connected to the catalytic layers. Nickel (Ni) is used at the anode, and platinum (Pt) at the cathode. The cell can be described with the following standard electrochemical notation:

$$\begin{array}{c} \left(\mathrm{Ni}\right)|H_{2}\;\left(p^{a},T^{a}\right),H_{2}O\;\left(p^{a},T^{a}\right)||O^{2-}||O_{2}\;\left(p^{c},T^{c}\right)|\left(\mathrm{Pt}\right). \end{array}$$

The upper script describes the anode (a) or the cathode (c). We define *y* as the spatial coordinate perpendicular to the active surface of the SOFC. The relevant molar fluxes $J_{i}$ are defined according to their flow direction, and the heat fluxes $J_{q}$ are defined as positive in the direction of the *y* axis. The flux $J_{q}^{a}\left(y\right)$ describes the heat through the homogeneous anode side GDL, while the heat flux occurring at the reaction layer is defined as $J_{q}^{a}\left(\Delta y^{a}\right)=J_{q}^{d,a}$. The heat flux $J_{q}^{e}\left(\Delta y^{a}\right)=J_{q}^{e,a}$, which has been embossed by the half-cell reaction, flows from the anode reaction layer into the electrolyte. The definition for the cathode side is analogous. The direction of the electric current density *j* is assumed to be the technical direction of the current against the electron flow. Throughout the entire simulations, an equimolar mixture of hydrogen and water on the anode side and air on the cathode side is supplied. Ideal gas mixtures are assumed. The thermodynamic reference state point is set to be at T=1123.15 K and p=1 bar.

### 2.2. Theory

#### 2.2.1. Mass and Energy Balance Equations for the Gas Diffusion Layers (GDL)

In the steady state, some component flow densities and the electric current density are in a fixed relationship to each other. The following relationships result from a steady-state component and charge balance:

(6) $$\begin{array}{c} 2\cdot J_{H_{2}}^{a}=-2\cdot J_{H_{2}O}^{a}=4\cdot J_{O_{2}}^{c}=j/F. \end{array}$$

$J_{k}^{i}$ describes the flux of each component, *j* is the electric current density, and F is the Faraday constant. The steady-state energy balance for the GDL of the anode and cathode side of an in *y*-direction infinitesimal volume element reads for the positive *y* direction:

$$\begin{array}{cc} \;\mathrm{Anode}: & \;0=\frac{dJ_{q}^{a}}{dy}+j\frac{d\phi }{dy}+J_{H_{2}}^{a}\frac{dH_{m,H_{2}}}{dy}-J_{H_{2}O}^{a}\frac{dH_{m,H_{2}O}}{dy}\\ \;\mathrm{Cathode}: & \;0=\frac{dJ_{q}^{c}}{dy}+j\frac{d\phi }{dy}-J_{O_{2}}^{c}\frac{dH_{m,O_{2}}}{dy}. \end{array}$$

Heat $J_{q}^{i}$, electric power j·dϕ, and the gaseous components *i* with a molar enthalpy $H_{m},i$ are supplied to or removed from the GDL. As ideal gas behavior is assumed in our approach, and no enthalpy of mixing has to be considered. If the relationship from the component balance is used, the energy balances result in:

(7) $$\begin{array}{cc} \;\mathrm{Anode}: & \;0=\frac{dJ_{q}^{a}}{dy}+j\left[\frac{d\phi }{dy}+\frac{1}{2F}\left[C_{m,p,H_{2}}^{\mathrm{iG}}\left(T\right)+C_{m,p,H_{2}O}^{\mathrm{iG}}\left(T\right)\right]\frac{dT}{dy}\right], \end{array}$$

(8) $$\begin{array}{cc} \;\mathrm{Cathode}: & \;0=\frac{dJ_{q}^{c}}{dy}+j\left[\frac{d\phi }{dy}+\frac{1}{4F}C_{m,p,O_{2}}^{\mathrm{iG}}\left(T\right)\frac{dT}{dy}\right]. \end{array}$$

The molar isobaric heat capacities $C_{m,p,i}^{\mathrm{iG}}\left(T\right)$ are calculated using a power series approach according to Kabelac et al. [21].

#### 2.2.2. Transport Equations for the Homogeneous Phase

Based on Equation (1), the general transport equations for the homogeneous phases are [20]:

(9) $$J_{q}^{i}=-L_{\mathrm{qq}}\;\frac{1}{T^{2}}\;\frac{dT}{dy}-\sum_{k=1}^{N}\;L_{q\mu k}\;\frac{1}{T}\;\frac{d\mu_{k,T}}{dy}-L_{q\phi }\;\frac{1}{T}\;\frac{d\phi }{dy},$$

(10) $$J_{k}=-L_{\mu_{k}q}\;\frac{1}{T^{2}}\;\frac{dT}{dy}-\sum_{j=1}^{N}\;L_{\mu_{k}\mu_{j}}\;\frac{1}{T}\;\frac{d\mu_{j,T}}{dy}-L_{\mu_{k}\phi }\;\frac{1}{T}\;\frac{d\phi }{dy},$$

(11) $$j=-L_{\phi q}\;\frac{1}{T^{2}}\;\frac{dT}{dy}-\sum_{k=1}^{N}\;L_{\phi \mu_{k}}\;\frac{1}{T}\;\frac{d\mu_{k,T}}{dy}-L_{\phi \phi }\;\frac{1}{T}\;\frac{d\phi }{dy},$$

with the local thermodynamic temperature T=T(y), the chemical potential $\mu_{k}$ of component *k*, and the chemical potential $\mu_{j}$ of component j≠k.

Unfortunately, the phenomenological coefficients $L_{\mathrm{ij}}$ are not well known at this point, and only some measurements of these special coefficients have been performed. They differ from the well-known coefficients from the empirical transport equations, but some helpful relations can be established using the following definition for an SOFC [20]:

(12) $$\mathrm{Thermal}\;\mathrm{conductivity}:\lambda =-{\left[\frac{J_{q}^{i}}{\left(dT/dy\right)}\right]}_{J_{k}=0,j=0},$$

(13) $$\mathrm{Diffusion}\;\mathrm{coefficient}:D_{k}=-{\left[\frac{J_{k}}{\left(dc_{k}/dy\right)}\right]}_{dT=0,j=0},$$

(14) $$\mathrm{Electrical}\;\mathrm{resistance}:r=-{\left[\frac{\left(d\phi /dy\right)}{j}\right]}_{dT=0,d\mu =0}g,$$

(15) $$\mathrm{Peltier}\;\mathrm{coefficient}:\pi =F\;{\left[\frac{J_{q}^{i}}{j}\right]}_{dT=0,d\mu =0},$$

(16) $$\mathrm{Thermal}\;\mathrm{diffusion}\;\mathrm{coefficient}:D_{T}=-{\left[\frac{J_{k}}{c_{k}\left(dT/dy\right)}\right]}_{dc_{k}=0,j=0}.$$

Very much attention has to be paid to the state variables which have to be constant for these relations. For an SOFC, simplified transport equations result if ideal gas mixture assumptions are made:

(17) $$J_{q}^{i}=-\lambda^{i}\;\frac{dT}{dy}+\frac{\pi^{i}}{F}\cdot j,$$

(18) $$J_{k}=-D_{T}\cdot c_{k}\;\frac{dT}{dy}-D_{k}\;\frac{dc_{k}}{dy},$$

(19) $$j=-\frac{\pi^{i}}{F\cdot r^{i}\cdot T}\frac{dT}{dy}-\frac{1}{r^{i}}\frac{d\phi }{dy}.$$

The electrical resistance $r^{i}$ is calculated via an Arrhenius approach according to Hajimolana et al. using the pre-exponential factor $r^{i},0$ [22]:

(20) $$r^{i}/T=r^{i,0}\cdot e^{\frac{E_{A,r}^{i}}{R_{m}T}}.$$

To calculate the Peltier coefficient, the entropy flow within the homogeneous phase *i* is derived according to NET, as follows, which, of course, also includes the local entropy production [20,23]:

(21) $$J_{s}=\frac{J_{q}^{i}}{T}+\sum_{k=1}^{n-1}J_{k}\cdot S_{m},k.$$

With the correlation from Equation (15), obeying the conditions dT=0, $d\mu_{i,T}=0$ and the assumption that no electroosmosis takes place, the following equation holds true for the Peltier coefficient:

(22) $$\pi =T\;{\left[\frac{J_{s}}{j/F}-\sum_{k=1}^{n-1}t_{k}\cdot S_{m,k}\right]}_{dT=0,d\mu_{i},T=0}=T\cdot \sum_{k=1}^{n-1}J_{k}\cdot S_{m},k,$$

with $S_{m,k}$ as the transported molar entropy of all n−1 components. The *n*-th component is chosen as the reference of frame. In this case, this is the positive ion lattice of the electrolyte as an unmoved reference wall, so that, for the anode and cathode reaction layer, the following equations result:

(23) $$\begin{array}{cc} \mathrm{Anode}: & \;\pi^{a}=T\cdot \left[\frac{1}{2}\;S_{m,H_{2}}-\frac{1}{2}\;S_{m,H_{2}O}+S_{m,e^{-},\mathrm{Ni}}\right], \end{array}$$

(24) $$\begin{array}{cc} \mathrm{Cathode}: & \;\pi^{c}=T\cdot \left[-\frac{1}{4}\;S_{m,O_{2}}+S_{m,e^{-},\mathrm{Pt}}\right]. \end{array}$$

The molar entropy $S_{m,k}$ of component *k* at temperature *T* is calculated by the entropic equation of state of ideal gases with the approach according to Kabelac et al. [21] for the molar isobaric heat capacities. The entropies of electrons in platinum and nickel are taken from References [24,25]. Approaches for determining the diffusion coefficient $D_{k}$ and the thermal diffusion coefficient $D_{T}$ are presented in the following sections.

The local entropy production rate of a homogeneous phase can now be estimated from Equation (2) as follows:

(25) $${\dot{\sigma }}^{i}=-J_{q}^{i}\;\frac{1}{T^{2}}\frac{dT}{dy}-j\;\frac{1}{T}\frac{d\phi }{dy}-R_{m}\sum_{k}J_{k}\frac{1}{c_{k}}\frac{dc_{k}}{dy}.$$

#### 2.2.3. Modeling of the Gas Diffusion Layers (GDL)

Fick’s law can be derived by simplifying assumptions from the more general Stefan–Maxwell diffusion and, thus, represents the simplest of the diffusion modeling. For the description and rating of a more complete and real mass transport modeling in the GDLs, Stefan–Maxwell diffusion (1) is described first and then overlaid with Knudsen diffusion (2), convection (3), and thermal diffusion (4). For a comparison of all these approaches, the mass transport is additionally modeled with Fick’s law. The Fick’s diffusion coefficients are calculated from the Knudsen diffusion coefficients, and the binary diffusion coefficients using the Bosanquet formula [16,26]:

(26) $$D_{k}^{\mathrm{eff}}={\left(\frac{1}{{Đ}_{\mathrm{kj}}^{\mathrm{eff}}}+\frac{1}{D_{k}^{\mathrm{Kn},\mathrm{eff}}}\right)}^{-1}.$$

Krishna et al. [26] state that, for molecules, such as H${}_{2}$, the Bosanquet formula is applicable to a wide range of pore diameters with reasonable accuracy. Since porous structures are assumed, some parameters, such as the permeability $B_{0}^{i}$ and the diffusion coefficients *D*, must be corrected by the porosity $\epsilon^{i}$, the tortuosity $\tau^{i}$, and the mean pore diameter $d_{p}^{i}$.

The NET approach according to Equation (10), respectively, Equation (18), represents another possibility for modeling the GDLs, and it is subsequently compared directly with the Dusty-Gas model (DGM) (1)–(4) described previously.

#### 2.2.4. Mass Transport by Stefan–Maxwell Diffusion (1)

If a mixture is considered, a diffusive movement relative of the individual components can occur. This relative movement leads to collisions between the different molecules. The driving force, caused by a gradient in the partial pressure of a component *k*, is in equilibrium with the frictional force, which is caused by the intermolecular collisions [27]. The equilibrium between the driving force and the frictional force in an ideal gas mixture of *N* components in a porous structure can be described by the following equation:

(27) $$-\frac{1}{R_{m}T}\frac{dp_{k}}{dy}=\sum_{j=1}^{N}\frac{x_{j}J_{k}^{\mathrm{diff}}-x_{k}J_{j}^{\mathrm{diff}}}{{Đ}_{\mathrm{kj}}^{\mathrm{eff}}}.$$

For the binary diffusion coefficients, we have ${Đ}_{\mathrm{kj}}^{\mathrm{eff}}={Đ}_{\mathrm{jk}}^{\mathrm{eff}}$. For the calculation of the effective binary diffusion coefficients, the approach of Fuller et al. is used [28]:

(28) $$\frac{{Đ}_{\mathrm{kj}}^{\mathrm{eff}}}{{\mathrm{cm}}^{2}/s}=\frac{\epsilon^{i}}{\tau^{i}}\cdot \frac{0.00143{\left(\frac{T}{K}\right)}^{1.75}{\left[{\left(\frac{M_{k}}{g/\mathrm{mol}}\right)}^{-1}+{\left(\frac{M_{j}}{g/\mathrm{mol}}\right)}^{-1}\right]}^{1/2}}{\frac{p}{\mathrm{bar}}\sqrt{2}{\left[v_{k}^{1^{/}3}+v_{j}^{1/3}\right]}^{2}}.$$

Here, *v* are the dimensionless diffusion volumes of the two components in a binary mixture, which are tabulated, e.g., in the VDI Heat Atlas [29].

#### 2.2.5. Extension by Knudsen Diffusion (2)

In narrow pores, in which the mean free path length of the molecules is considerably greater than the pore diameter, there are increased collisions of the molecules with the pore wall [30]. Because of these collisions, the following gradient in partial pressure result for the individual component *k* according to Reference [27]:

(29) $$\frac{dp_{k}}{dy}=-R_{m}T\frac{J_{k}}{D_{k}^{\mathrm{Kn},\mathrm{eff}}}.$$

For the mass flow density of nitrogen, $J_{N_{2}}=0$ applies in a steady state operation, as this inert gas does not react at the reaction layers. The effective Knudsen diffusion coefficients $D_{k}^{\mathrm{Kn},\mathrm{eff}}$ of the individual components are calculated according to Krishna and Wesselingh [27] via the kinetic gas theory:

(30) $$D_{k}^{\mathrm{Kn},\mathrm{eff}}=\frac{\epsilon^{i}}{\tau^{i}}\cdot \frac{d_{p}^{i}}{3}\sqrt{\frac{8R_{m}T}{\pi M_{k}}}.$$

To superimpose the Stefan–Maxwell diffusion and the Knudsen diffusion, the partial pressure gradients of the two transport mechanisms may be added [27,30,31]. The idea follows exclusively from the additivity of the momentum transfer, without any theoretical proof [31]. The following transport equation results in a steady state operation:

(31) $$\frac{dp_{k}}{dy}=-R_{m}T\left(\sum_{j=1}^{N}\frac{x_{j}J_{k}^{\mathrm{diff}}-x_{k}J_{j}^{\mathrm{diff}}}{{Đ}_{\mathrm{kj}}^{\mathrm{eff}}}+\frac{J_{k}}{D_{k}^{\mathrm{Kn},\mathrm{eff}}}\right).$$

#### 2.2.6. Extension by Convection (3)

In convection, the gas mixture moves as a continuum and is driven by a gradient in the total pressure. Typically the adhesion condition applies to the walls of the flow channel from a macroscopic view, which means that the velocity of the viscous flow is zero directly at the wall interface. The convective mass flow density of the respective component can be calculated by Darcy’s law, with the permeability $B_{0}^{i}$ and the dynamic viscosity of the mixture η [27]:

(32) $$J_{k}^{\mathrm{konv}}=-x_{k}\frac{B_{0}^{i}p}{\eta R_{m}T}\frac{dp}{dy}.$$

The permeability $B_{0}^{i}$ depends on the geometry of the pores and must also be corrected due to the porous structure. For the effective permeability within circular open pores, Equation (33) applies [30]:

(33) $$B_{0}^{i,\mathrm{eff}}=\frac{\epsilon^{i}}{\tau^{i}}\frac{{\left(d_{p}^{i}\right)}^{2}}{32}.$$

The total mass flow density, which occurs in a steady state operation, results from the summation of the diffusive and convective component [27,30,31]:

(34) $$J_{k}=J_{k}^{\mathrm{diff}}+J_{k}^{\mathrm{konv}}.$$

This results in the following transport equation, which, in addition to Knudsen and Stefan–Maxwell diffusion, also takes convection into account:

(35) $$\frac{dp_{k}}{dy}+\frac{B_{0}^{i,\mathrm{eff}}p_{k}}{\eta D_{k}^{\mathrm{Kn},\mathrm{eff}}}\frac{dp}{dy}=-R_{m}T\left(\sum_{j=1}^{N}\frac{x_{j}J_{k}-x_{k}J_{j}}{{Đ}_{\mathrm{kj}}^{\mathrm{eff}}}+\frac{J_{k}}{D_{k}^{\mathrm{Kn},\mathrm{eff}}}\right).$$

The dynamic viscosities of the individual gases $\eta_{k}^{\mathrm{iG}}$ are calculated using Lucas et al.’s correlation equation recommended in the VDI Heat Atlas, which is permissible due to the low operating pressure in the SOFC [29]. All quantities required for the calculation of the dynamic viscosities of the pure substances can be taken from Reference [29]. Subsequently, the dynamic viscosities of the gas mixtures η are calculated for both the anode and the cathode on the basis of the mass fractions and the dynamic viscosities of the pure substances via the mixing rule according to Wilke [32]:

(36) $$\eta =\sum_{k}\frac{x_{k}\eta_{k}^{\mathrm{iG}}}{\sum_{j}x_{j}F_{\mathrm{kj}}},$$

with

$$F_{\mathrm{kj}}=\frac{{\left[1+{\left(\eta_{k}^{\mathrm{iG}}/\eta_{j}^{\mathrm{iG}}\right)}^{1/2}{\left(M_{j}/M_{k}\right)}^{1/4}\right]}^{2}}{\sqrt{8(1+M_{k}/M_{j})}}.$$

#### 2.2.7. Extension through Thermal Diffusion (4)

Thermal diffusion, also called the Soret effect, is a coupled process, in which a flow of substances is caused by a temperature gradient. If the mixture is subject to a temperature gradient, gradients are established in the concentration of the individual components [33]. Heavy molecules arrange themselves more in the colder regions, and light molecules in the warmer regions. Thermal diffusion belongs to multi-component diffusion. Therefore, a version of the Stefan–Maxwell diffusion according to Krishna and Wesselingh, extended by the thermal diffusion rate, is used to consider thermal diffusion [27]:

(37) $$-\frac{1}{R_{m}Tc}\frac{dp_{k}}{dy}=\sum_{j=1}^{N}\frac{x_{k}x_{j}\left(w_{k}^{T}-w_{j}^{T}\right)}{{Đ}_{\mathrm{kj}}},$$

with

(38) $$w_{k}^{T}=w_{k}^{\mathrm{diff}}+D_{\mathrm{kj}}^{T}\frac{dT}{dy}.$$

The extended velocity $w_{k}^{T}$ is obtained by the summation of the diffusion velocity $w_{k}^{\mathrm{diff}}$ with the velocity due to thermal diffusion, which can be calculated by the thermal diffusion coefficient $D_{\mathrm{kj}}^{T}$. A positive thermal diffusion coefficient means that the component moves toward colder regions, and a negative coefficient means that the component moves toward warmer regions [27]. For the thermodiffusion coefficient, we have [31]:

(39) $$D_{\mathrm{kj}}^{T}=-D_{\mathrm{jk}}^{T}.$$

The thermal diffusion coefficients are often expressed by the thermal diffusion rate $k_{\mathrm{kj}}^{T}$ or the thermal diffusion factor $\alpha_{\mathrm{kj}}^{T}$ [34]:

(40) $$k_{12}^{T}=\frac{TD_{12}^{T}}{{Đ}_{12}}=\alpha_{12}^{T}x_{1}x_{2}.$$

Taking thermal diffusion into account, the general transport equation within the GDL results:

(41) $$\frac{dp_{k}}{dy}+\frac{B_{0}^{i,\mathrm{eff}}p_{k}}{\eta D_{k}^{\mathrm{Kn},\mathrm{eff}}}\frac{dp}{dy}=-R_{m}T\left(\sum_{j=1}^{N}(\frac{x_{j}J_{k}-x_{k}J_{j}}{{Đ}_{\mathrm{kj}}^{\mathrm{eff}}}+2c{\left(x_{k}x_{j}\right)}^{2}\frac{\alpha_{\mathrm{kj}}^{T}}{T}\frac{dT}{dy})+\frac{J_{k}}{D_{k}^{\mathrm{Kn},\mathrm{eff}}}\right).$$

From the equation, it can be seen that thermodiffusion can both improve or dampen mass transfer. This depends on the signs of the thermal diffusion factor and the temperature gradient. If a component moves against the expected transport direction as a result of the thermal diffusion, the thermal diffusion acts as an additional transport resistance that must be overcome by a higher partial pressure gradient.

The thermal diffusion factor $\alpha_{\mathrm{kj}}^{T}$ can be calculated by means of the kinetic theory of gases, which relates the macroscopic transport coefficients to the intermolecular interactions of gases [35]. A model that describes the intermolecular potential as a function of the distance between the molecular centers $r^{*}$ is the Lennard–Jones (12-6) potential $\phi^{\mathrm{LJ}}$. Data for the maximum attraction between molecules $\epsilon_{k}$ and for the characteristic Lennard-Jones length $\sigma_{k}$ are taken from Reference [36]. Widely used calculation approaches for the thermal diffusion factor $\alpha_{\mathrm{kj}}^{T}$, which are based on the kinetic gas theory, are the first two approximations according to Chapman and Cowling and the first approximation according to Kihara. The first approximation according to Kihara provides better results than the approaches of Chapman and Cowling [37], which is why it is used in this work [38]:

(42) $$\alpha_{12}^{T}=\left(\frac{S_{1}x_{1}-S_{2}x_{2}}{Q_{1}x_{1}^{2}+Q_{2}x_{2}^{2}+Q_{12}x_{1}x_{2}}\right)\left(6C_{12}^{*}-5\right).$$

Here, $S_{k}$ and $Q_{k}$ are functions of the molecular masses, the Lennard-Jones parameters, and the collision integrals which account for the different interactions between the individual molecules in a binary mixture. Additional collision integrals are summarized in $C_{12}^{*}$. These multi-dimensional collision integrals were calculated and tabulated in Reference [35] for different potential functions, depending on the temperature. By convention, the index 1 stands for the molecule with the greater mass.

#### 2.2.8. Mass Transfer Approach According to NET

Through comparison of the coefficients of Equations (10) and (18), it follows that, for the phenomenological coefficients:

(43) $$L_{\mu_{k}q}=D_{T}\cdot c_{k}\cdot T^{2}$$

and

(44) $$L_{\mu_{k}\mu_{k}}=\frac{D_{k}\cdot c_{k}}{R}.$$

The diffusion coefficient $D_{k}$ can be determined using Equation (26). The thermal diffusion coefficient $D_{T}$ is calculated via the approach of Equation (40).

#### 2.2.9. Modeling of the Reaction Layers

In this model, reactions are assumed to take place on the surfaces of the electrodes only. A homogeneous temperature profile is additionally assumed at the electrode surfaces. However, jumps in the y-direction occur in the course of the electrical potential and the heat flux density. The heat flux density is calculated via an entropy balance, including the reaction entropy. The material current densities are expressed by the electric current density. For the oxygen ion flux, $J_{O^{2-}}=-\frac{j}{2\cdot F}$ results.

(45) $$\begin{array}{cc} \mathrm{Anode}: & \;J_{q}^{e,a}=J_{q}^{d,a}-T\cdot \frac{j}{2F}\cdot \Delta^{R}S_{m}^{a}\left(T\right)+T\cdot {\dot{s}}_{\mathrm{irr}}^{a}, \end{array}$$

(46) $$\begin{array}{cc} \mathrm{Cathode}: & \;J_{q}^{d,c}=J_{q}^{e,c}-T\cdot \frac{j}{2F}\cdot \Delta^{R}S_{m}^{c}\left(T\right)+T\cdot {\dot{s}}_{\mathrm{irr}}^{c}, \end{array}$$

with

$$\Delta^{R}S_{m}^{a}\left(T\right)=S_{m,H_{2}O}\left(T\right)-S_{m,H_{2}}\left(T\right)-S_{m,O^{2-}}\left(T\right)+2\cdot S_{m,e^{-},\mathrm{Ni}}\left(T\right),$$

$$\Delta^{R}S_{m}^{c}\left(T\right)=S_{m,O^{2-}}\left(T\right)-\frac{1}{2}S_{m,O_{2}}\left(T\right)-2\cdot S_{m,e^{-},\mathrm{Pt}}\left(T\right).$$

The entropy of the oxygen ion $S_{O^{2-}}$ is calculated using the approach of Kjelstrup and Tomii [14].

The entropy production rates ${\dot{s}}_{\mathrm{irr}}^{i}$ result from the activation overvoltages at the respective electrode. Butler–Volmer kinetics is used to calculate the activation overvoltages:

(47) $${\dot{\xi }}_{r}^{i}=\frac{j_{0}^{i}}{2\cdot F}\left(e^{-\frac{\alpha^{i}\cdot 2\cdot F}{R_{m}\cdot T}\cdot \eta^{i,\mathrm{act}}}-e^{\frac{(1-\alpha^{i})\cdot 2\cdot F}{R_{m}\cdot T}\cdot \eta^{i,\mathrm{act}}}\right).$$

A steady-state charge balance yields $j=2\cdot F\cdot \xi_{\mathrm{HOR}}^{a}$ for the anode and $j=-2\cdot F\cdot \xi_{\mathrm{ORR}}^{c}$ for the cathode. The exchange current densities are determined by an Arrhenius approach according to Yonekura et al. [39]:

(48) $$j_{0}^{a}=\left[{\left(x_{H_{2}}^{R}\right)}^{0.41}{\left(x_{H_{2}O}^{R}\right)}^{0.4}\right]\gamma^{a}\cdot e^{-\frac{E_{A,j_{0}}^{a}}{R_{m}T}},$$

(49) $$j_{0}^{c}={\left(x_{O_{2}}^{R}\right)}^{0.3}\gamma^{c}\cdot e^{-\frac{E_{A,j_{0}}^{c}}{R_{m}T}},$$

where the pre-exponential factors $\gamma^{a}$ and $\gamma^{c}$. The activation energies $E_{A,j_{0}}^{i}$ have been determined empirically by electrochemical impedance spectroscopy in Reference [12]. Using the activation overvoltages, the entropy production rates are finally calculated as:

(50) $$\begin{array}{cc} {\dot{s}}_{\mathrm{irr}}^{a}= & \eta^{a,\mathrm{act}}\cdot j/T, \end{array}$$

(51) $$\begin{array}{cc} {\dot{s}}_{\mathrm{irr}}^{c}= & -\eta^{c,\mathrm{act}}\cdot j/T. \end{array}$$

The potential curve at the reaction boundary layer is calculated as:

(52) $$\begin{array}{cc} \phi^{e,a}= & \phi^{d,a}-\Delta \phi_{0}^{a}\left(p_{k}^{R}\right)-\eta^{a,\mathrm{act}}, \end{array}$$

(53) $$\begin{array}{cc} \phi^{c,d}= & \phi^{e,c}+\Delta \phi_{0}^{c}\left(p_{k}^{R}\right)+\eta^{c,\mathrm{act}}. \end{array}$$

Thus, the potential immediately behind the reaction boundary layer is calculated from the potential immediately before the reaction boundary layer, the electrode potentials in no-load operation, and the activation overvoltages. As the equilibrium electrode potentials are calculated with the reactant concentrations in the reaction zones, a determination of the concentration overvoltage $\eta^{i,\mathrm{con}}$ is not necessary. The electrode potentials in an open circuit situation at the individual electrodes are obtained by applying the equilibrium condition of an electrochemical reaction and correspond to $\Delta \phi_{0}^{i}=\left({\tilde{\mu }}_{O^{2-}}-2{\tilde{\mu }}_{e^{-}}\right)/2\cdot F$ [12]. The electrode potentials are given by the following equations:

(54) $$\begin{array}{cc} \Delta \phi_{0}^{a}\left(x_{k}^{R}\right)= & \frac{1}{2F}\left[G_{m,H_{2}O}^{\theta }\left(T\right)-G_{m,H_{2}}^{\theta }\left(T\right)+R_{m}T\cdot \ln\left(\frac{x_{H_{2}O}^{R}}{x_{H_{2}}^{R}}\right)\right], \end{array}$$

(55) $$\begin{array}{cc} \Delta \phi_{0}^{c}\left(x_{k}^{R}\right)= & \frac{1}{2F}\left[0,5\cdot G_{m,O_{2}}^{\theta }\left(T\right)+\frac{R_{m}T}{2}\ln\left(x_{O_{2}}^{R}\right)\right]. \end{array}$$

#### 2.2.10. Modeling of the Electrolyte

In an SOFC electrolyte, the diffusion of the oxygen ions leads not only to a mass transport but also to a proportional charge transport. This relationship is explained by the definition of the electrochemical potential ${\tilde{\mu }}_{O^{2-}}=\mu_{O^{2-}}-z_{O^{2-}}^{*}\cdot F\cdot \psi$ according to Guggenheim [40], where $\mu_{O^{2-}}$ is the chemical potential of the anion, $z_{O^{2-}}^{*}=2$ is the charge number of the anion, and ψ is the electrostatic potential. Taking into account the charge conservation law and the assumptions that no cation diffusion occurs in YSZ at 1300 K and polarization effects can become negligible, we obtain [12]:

(56) $$\frac{j}{z_{O^{2-}}^{*}\cdot F}=J_{O^{2-}}^{e}.$$

with Equation (56) and the relation

(57) $$\phi =\frac{\mu_{O^{2-}}}{z_{O^{2-}}^{*}\cdot F}-\psi ,$$

according to Equations (12)–(15), the following transport equations apply to the electrolyte [12,41]:

(58) $$\begin{array}{cc} J_{q}^{e} & =-L_{\mathrm{qq}}\;\frac{1}{T^{2}}\;\frac{dT}{dy}-L_{\mathrm{qO}}\;\frac{1}{T}\;\frac{d}{dy}\left[\mu_{O^{2-}}-z_{O^{2-}}^{*}\cdot F\cdot \psi \right] \end{array}$$

(59) $$=-L_{\mathrm{qq}}\;\frac{1}{T^{2}}\;\frac{dT}{dy}-L_{\mathrm{qO}}\;\frac{z_{O^{2-}}^{*}\cdot F}{T}\;\frac{d\phi }{dy},$$

(60) $$\begin{array}{cc} J_{O^{2-}}^{e} & =-L_{\mathrm{Oq}}\;\frac{1}{T^{2}}\;\frac{dT}{dy}-L_{\mathrm{OO}}\;\frac{1}{T}\;\frac{d}{dy}\left[\mu_{O^{2-}}-z_{O^{2-}}^{*}\cdot F\cdot \psi \right] \end{array}$$

(61) $$\begin{array}{cc} \; & =-L_{\mathrm{Oq}}\;\frac{1}{T^{2}}\;\frac{dT}{dy}-L_{\mathrm{OO}}\;\frac{z_{O^{2-}}^{*}\cdot F}{T}\;\frac{d\phi }{dy}, \end{array}$$

(62) $$\begin{array}{cc} j & =-L_{\psi q}\;\frac{F}{T^{2}}\;\frac{dT}{dy}-L_{\psi \psi }\;\frac{F}{T\cdot z_{O}^{*}2-}\;\frac{d}{dy}\left[\mu_{O^{2-}}-z_{O^{2-}}^{*}\cdot F\cdot \psi \right] \end{array}$$

(63) $$=-L_{\psi q}\;\frac{F}{T^{2}}\;\frac{dT}{dy}-L_{\psi \psi }\;\frac{F^{2}}{T}\;\frac{d\phi }{dy},$$

with ϕ=ϕ(y) as the electric potential. By the relation from Equation (56), two of three transport equations are independent of each other, which completely describe the coupled transport of heat and oxygen ions. The transport equations for the electrolyte finally result as a function of the empirical coefficients:

(64) $$\begin{array}{cc} J_{q}^{e}= & -\lambda^{e}\;\frac{dT}{dy}+\frac{\pi^{e}}{F}\cdot j, \end{array}$$

(65) $$\begin{array}{cc} j= & -\frac{\pi^{e}\cdot \sigma^{e}}{F}\frac{1}{T}\frac{dT}{dy}-\sigma^{e}\;\frac{d\phi }{dy}. \end{array}$$

For the Peltier coefficient in the electrolyte, Equation (22) applies:

(66) $$\pi^{e}=-\frac{1}{2}\;S_{O^{2-}}T.$$

The gradient of the heat flux density is determined from an energy balance in a time independent state:

(67) $$0=\frac{dJ_{q}^{e}}{dy}+J_{O^{2-}}^{e}\cdot z_{O^{2-}}^{*}\cdot F\;\frac{d\phi }{dy}.$$

#### 2.2.11. Exergy Analysis

For a full evaluation of the efficiency of the system, the exergetic efficiency ζ is used. Exergy $\dot{E}x$ is a thermodynamic state variable, and it describes the part of energy that can be converted into any other form of energy. An energy conversion dissipates energy; so, some exergy $\dot{E}x$ is converted to anergy $\dot{B}$. This fraction is called exergy loss $\dot{E}x_{L}$. In this dissipative process, anergy cannot be converted into any other form of energy. For the exergetic efficiency of a fuel cell, the fraction of the useful outgoing exergy to the incoming exergy is:

(68) $$\begin{array}{c} \zeta =\frac{U\cdot j}{{\dot{n}}^{a}\cdot \left(\dot{E}x_{\mathrm{ph}}^{a}+\dot{E}x_{\mathrm{ch}}^{a}\right)+{\dot{n}}^{c}\cdot \left(\dot{E}x_{\mathrm{ph}}^{c}+\dot{E}x_{\mathrm{ch}}^{c}\right)}, \end{array}$$

with $\dot{E}x_{\mathrm{ph}}^{i}$ as physical exergy of a material flow [42]:

(69) $$\begin{array}{c} \dot{E}x_{\mathrm{ph}}^{i}={\dot{n}}^{i}\cdot [H_{m}\left(T,p,x_{i}\right)-H_{m}\left(T_{0},p^{\theta },x_{i}\right)-T_{0}\cdot \left(S_{m}\left(T,p,x_{i}\right)-S_{m}\left(T_{0},p^{\theta },x_{i}\right)\right], \end{array}$$

and $\dot{E}x_{\mathrm{ch}}^{i}$ as chemical exergy of a material flow [42]:

(70) $$\dot{E}x_{\mathrm{ch}}^{i}={\dot{n}}^{i}\cdot \left[\sum_{k=1}^{N}x_{k}\cdot Ex_{m}^{\theta },k\left(T_{0}\right)+R\cdot T_{0}\cdot \sum_{k=1}^{N}x_{k}\cdot \ln x_{k}\right].$$

The equations are valid for ideal gas mixtures. The chemical exergy of a substance $Ex_{m,k}^{\theta }\left(T_{0}\right)$ at $T_{0}=298.15$ K can be taken from Reference [43].

### 2.3. Simulation

The model equations derived in the previous section are used to calculate one-dimensional profiles of partial pressures, temperature, heat flux, electric potential, and local entropy productions rate. MATLAB^®^ version R2020a software is used to perform these calculations. The operating conditions of the cell are fixed, which include the operating temperature *T*, the operating pressure *p*, the composition of the inlet gases, and the electric current density *j*. These operating conditions also determine the boundary conditions of the cell. The cell temperature agrees with the operating temperature, both when y=0 and when $y=\Delta y^{a}+\Delta y^{e}+\Delta y^{c}$. At the anode side of the cell, the electric potential ϕ(y=0) is set to zero. The other boundary conditions are determined by the partial pressures of the inlet gases. As start values for the iteration, the heat flux density $J_{q}\;\left(y=0\right)$ and the partial pressures $p_{O_{2}}^{R}$ and $p_{N_{2}}^{R}$ of the cathode gases in the reaction zone are estimated first. The differential equation systems for the anode GDL, the electrolyte, and the cathode GDL, which result from the transport and balance equations, are solved step by step using the Runge–Kutta method. The profiles at the reaction layers are calculated using the Equations from Section 2.2.9. If the boundary conditions are not met after the first calculation round, the starting values are varied, and the cell is calculated again. The starting values are then adjusted using Newton’s method to start the next iteration step.

#### 2.3.1. General Parameters

The parameters for a specific example case are summarized in Table 1. The dimensions of the modeled SOFC single cell correspond to a type KeraCell III from the company Kerafol GmbH (Eschenbach i. d. Opf., Germany) [44]. The validation is carried out using the known U,j characteristic curve of the cell under consideration. The U,j-characteristic curve determined experimentally by the manufacturer can be taken from the data sheet. Furthermore, a U,j-characteristic curve was recorded at the Institute with an existing SOFC/SOEC test bench (Evaluator C1000-HT) from HORIBA FuelCon (Magdeburg-Barleben, Germany) under the same conditions.

For an electrolyte of YSZ08, many references exist in which the thermal conductivity or the ionic conductivity were determined experimentally. In the publications by Schlichting et al. [45], Sasaki et al. [46], and Radovic et al. [47], measured thermal conductivities are presented as a function of temperature. Here, the thermal diffusivity *a* is measured using laser flash analysis. The density of the test specimen is determined by Schlichting et al. using Archimedes’ principle. In the work of Sasaki et al., the mass and volume of the test specimen are measured in order to subsequently calculate the density. Radovic et al. do not make any statements about the measurement of the density. The specific heat capacity *C* of the ceramic is measured in all three papers using dynamic differential calorimetry. Sasaki et al. determine the thermal diffusivity of a non-porous ceramic by linear extrapolation of the values of a porous ceramic. The ionic conductivity of YSZ08 was measured experimentally in the work of Gibson and Irvine [48], Antunes et al. [49], and Artemov et al. [50]. In these papers, the impedance spectrum of the ceramic is measured at different temperatures using impedance spectroscopy to subsequently determine the ionic conductivity. Similar results are obtained in all three papers. Due to the larger data range, the data of Gibson and Irvine are chosen for our modeling.

#### 2.3.2. Parameters for Mass Transport

For a detailed investigation of the influence of different mass transfer equations, the mass flow of all individual components are plotted as a function of the current density in the anode and cathode GDL. The required parameters and their origin can be found in Table 2.

## 3. Results and Discussion

### 3.1. Validation

**Figure 2:**
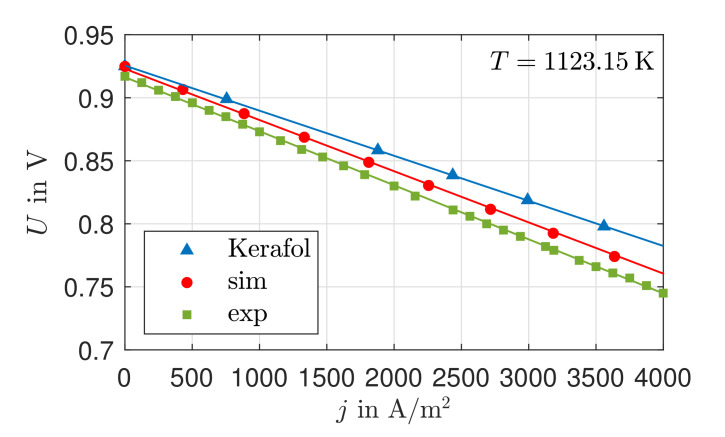
U,j-characteristics of an SOFC of the type KeraCell III with an electrolyte of 8YSZ at T=1123.15 K.

Figure 2 shows our own experimentally determined (exp) and the simulated (sim) U,j-characteristic curves for T=1123.15 K, as well as values from Kerafol GmbH (Kerafol). The simulated open circuit voltage (OCV) is 0.08% lower compared to the value of Kerafol. With increasing current density, the deviation grows until it reaches a value of 0.78% at $j=4000\;A/m^{2}$. Reasons for this are the calculation of the losses in the reaction layers and/or in the electrolyte. The dominance of the ohmic losses is due to the limited ionic conductivity of solid oxide ceramics and the strong dependence of the ohmic losses on the electric current density. From the calculation of the overvoltages at the electrodes due to the reaction kinetics, high exchange current densities occur due to the high operating temperature. However, in contrast to the ohmic losses, there is no linear relationship to the increasing electric current density. For this reason, it is assumed that slightly increased ohmic losses are assumed in the model.

The deviation of the simulated OCV is 0.84% compared to the experimentally determined value. This can be explained by the leakages occurring in the experiments and slightly varying gas concentrations. With increasing current density, the deviations also increase. At a current density of $j=4000\;A/m^{2}$, there is a deviation of 0.93%. The deviations can be explained by several factors. These include activation and concentration overvoltages at the electrodes, charge transport losses across the electrolyte, and ohmic resistances in the electrical contacts. The deviations of the model given are within a reasonable range, such that further investigations will be continued with the parameters present.

### 3.2. Partial Pressures

**Figure 3:**
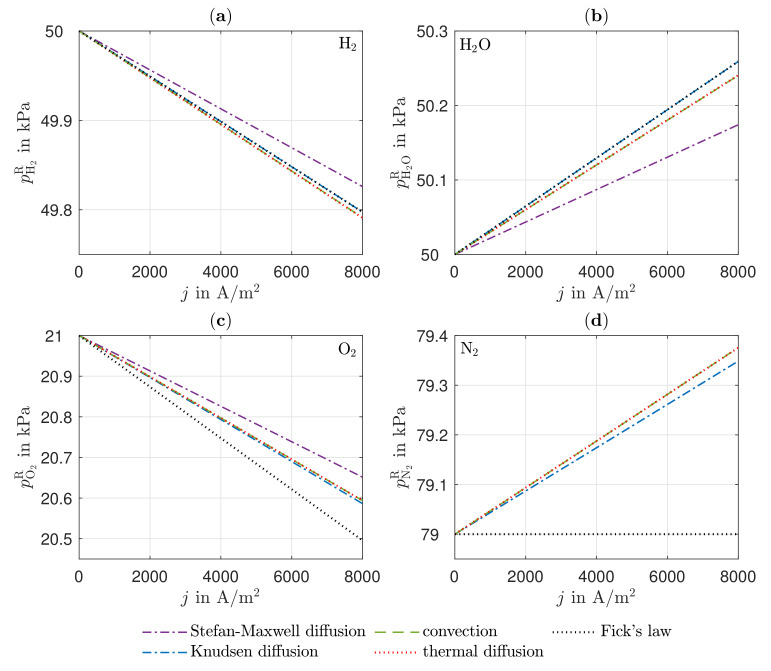
Partial pressure of (**a**) hydrogen and (**b**) water in the anode reaction layer, (**c**) oxygen and (**d**) nitrogen in the cathode reaction layer, as a function of the electric current density *j*.

In Figure 3, the partial pressures of all components in the reaction layers are shown as a function of the electric current density at T=1123.15 K. The calculated various diffusion coefficients are summarized in Table 3. The Stefan–Maxwell diffusion and Fick’s law graphs are described as pure mass transport for better comparability. All further graphs include the previously implemented mechanism.

With increasing electric current density, the chemical reaction rates also increase, resulting in a decrease in the partial pressure of the hydrogen on the anode side and an increase in the partial pressure of water. Due to the same effective binary diffusion coefficient, the curves of the pure Stefan–Maxwell diffusion show the same slopes in terms of magnitude. Furthermore, the graphs for the Knudsen diffusion—Fick’s law and convection—thermal diffusion overlap. Since Knudsen diffusion additionally takes into account the collisions of the molecules with the pore wall, there is a significant difference to pure Stefan–Maxwell diffusion. The overlaid Knudsen diffusion shows hardly any noticeable differences in the partial pressures compared to Fick’s law, although Fick’s law does not take into account the force of friction caused by the intermolecular collisions. The mass flow densities of the hydrogen and the water are the same due to the equimolar composition, which means that no differences are apparent in the superimposed Knudsen diffusion and Fick’s law. However, the different components are affected differently by Knudsen diffusion. On the one hand, this is due to the different molecular sizes of the components, which results in the largest effective Knudsen diffusion coefficient for hydrogen. Thus, the effect of Knudsen diffusion on the mass transport of hydrogen is the smallest. As the partial pressure gradients of the individual components are not equal on both the anode and cathode sides due to the different effect of Knudsen diffusion, a gradient is established in the total pressure, which leads to some additional convection of the gas mixtures. However, it can be seen that the effect of convection is small in comparison to the other transport mechanisms, although all gases have low dynamic viscosities. The reason for this lies in the low gradients in the total pressure. In the anode reaction layer, there is a higher total pressure than in the gas channel, which is why the gas mixture moves in the direction of the gas channel as a result of the convection, thus impeding the mass transport of the hydrogen and improves the mass transport of the water. Thermal diffusion shows no influence on mass transfer under the operating conditions considered. The reason for this is that the possible prevailing temperature gradients are too small to give thermal diffusion a significant influence. The temperature gradients in the anode, electrolyte, and cathode are explained in more detail in the following sections.

The partial pressure of the nitrogen remains unchanged in the steady state due to $J_{N_{2}}=0$. For this reason, the partial pressure curves of the Stefan–Maxwell and Knudsen diffusion also overlap. Since the total pressure in the gas channel is higher in the cathode than in the reaction layer, convection improves the oxygen transport. This leads to a larger gradient in the partial pressure of nitrogen. These partial pressure gradients of nitrogen are necessary to counteract the drag force due to the molecular collisions with the oxygen and the convection, thus ensuring $J_{N_{2}}=0$. The influence of Stefan–Maxwell diffusion on the cathode gases is greater, since a clearly smaller effective binary diffusion coefficient results for the cathode-side gas mixture. As oxygen is the largest of the molecules present, it has the lowest effective Knudsen diffusion coefficient. However, the effect of the superimposed Knudsen diffusion is smaller than for water due to the lower mass flow density of oxygen. The improved mass transfer of oxygen due to convection is very small, as the gas mixture consists mostly of nitrogen on the cathode side. The mass transport via Fick’s law shows the greatest changes in the gradient of the partial pressure for oxygen. By taking into account the mole fraction of oxygen in the Stefan–Maxwell diffusion, the mass transport is improved by a significant amount. On the cathode side, thermal diffusion also has no influence on mass transport under the operating conditions considered here.

**Figure 4:**
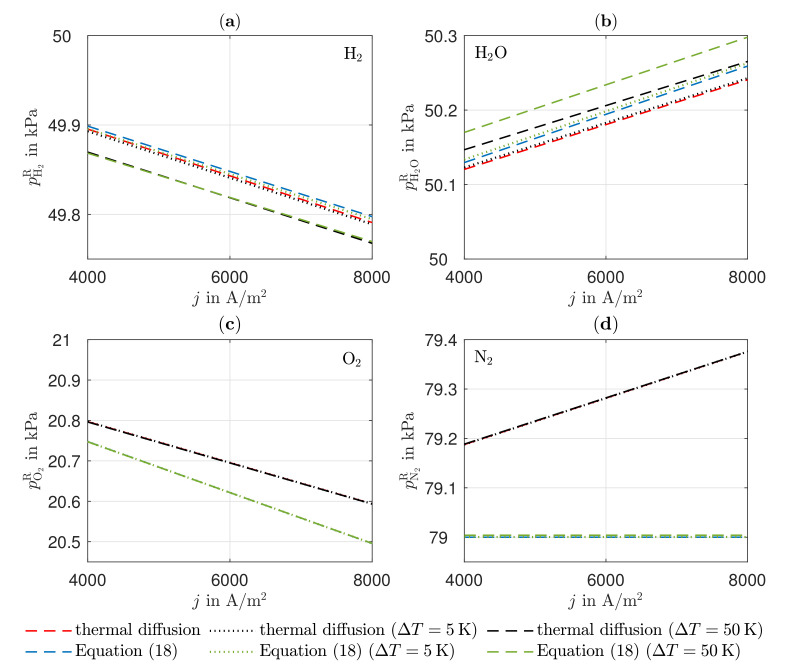
Partial pressure of (**a**) hydrogen and (**b**) water in the anode reaction layer, (**c**) oxygen and (**d**) nitrogen in the cathode reaction layer, as a function of the electric current density j for different imposed temperature gradients ΔT between cathode and anode.

Figure 4 shows the partial pressure profiles of the gas components, determined via the thermal diffusion approach according to Section 2.2.7, and the TiP approach according to Section 2.2.8. In the anode, the partial pressures of hydrogen and water determined by the TiP approach behave as the superimposed Knudsen diffusion, as the thermal diffusion approach includes the extension to convection. Due to the prevailing low temperature gradients, the thermodiffusion in the TiP approach according to Equation (10) does not take influence on the partial pressure curves. Even imposing a temperature gradient of ΔT=5 K over the electrolyte thickness $\Delta y^{e}$ shows a change in the range of $10^{-}3$ kPa only. Only by imposing ΔT=50 K over $\Delta y^{e}$ can two essential facts be clarified. First, the mass transfer for both components is worsened by imposing of a temperature gradient, i.e., the effect of thermal diffusion. Secondly, the consideration of convection is highlighted, which worsens the mass transfer of hydrogen and improves that of water. The differences between the two approaches can be explained using Equations (18) and (40). In Equation (40), the thermal diffusion coefficient is divided by the binary diffusion coefficient, whereas, in Equation (18), the thermal diffusion coefficient is divided by the binary and Knudsen diffusion coefficients after rearranging the equation. As the Knudsen diffusion coefficient of hydrogen is higher than that of water, the term has a smaller effect on hydrogen, so that smaller differences are seen in the partial pressure of hydrogen between the two approaches. Due to the very small thermal diffusion coefficients of oxygen and nitrogen, thermal diffusion does not affect mass transfer despite the imposition of a temperature gradient (cf. Figure 4). The similar molar masses of oxygen and nitrogen result in small thermodiffusion factors, which means that mass transfer of a mixture of these two components due to a temperature gradient has no effect.

### 3.3. Heat Transport, Temperature Gradient, and Potential Field

**Figure 5:**
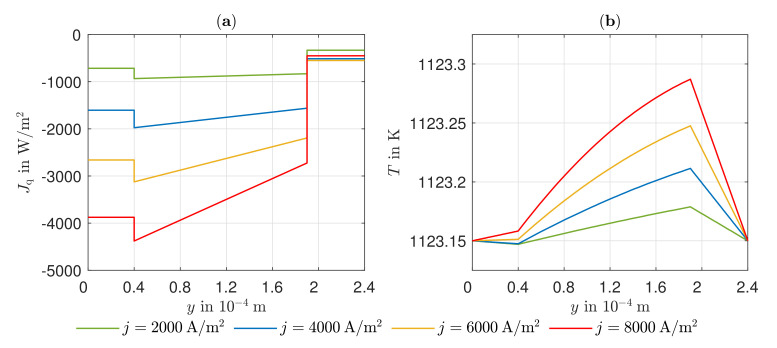
Simulated profile of (**a**) heat flux density and (**b**) temperature along spatial coordinate *y*.

Figure 5 shows the simulated heat flux density curves and the temperature fields at T=1123.15 K for different electric current densities as a function of the spatial coordinate y. It can be seen that the heat flow $J_{q}$ always has a negative sign, i.e., heat flows from the cathode toward the anode. The jumps in the course of the heat flow at the reaction layers follow, on the one hand, from the irreversible activation overvoltages. In addition, at the cathode, heat is released due to the negative reaction entropy ($\Delta^{R}S_{m}^{c}\left(1123.15\;K\right)=-80.47\;J/\mathrm{mol}\;K$), whereas the anode consumes heat due to the positive reaction entropy ($\Delta^{R}S_{m}^{a}\left(1123.15\;K\right)=21.65\;J/\mathrm{mol}\;K$). This leads to a big amount of heat being released at the cathode due to these two effects. Net heat is absorbed at the anode because, here, the heat consumption due to the reaction entropy exceeds the heat released due to the irreversibilities. Thus, in the steady state, there is an increased temperature at the cathode and a decreased temperature at the anode. Within the electrolyte, the heat generated due to irreversible ion transport can be recognized by heat flux densities increasing in magnitude in the direction of the charge transport. Considering the temperature curve along the spatial coordinate, the relevance of the Peltier effects in the cell becomes clear. At the GDL of the cathode, the heat flow flows from a lower to a higher temperature accordingly. At the GDL of the anode, the Peltier effect shows a smaller impact due to the smaller coefficient ($\pi^{a}\left(1123.15\;K\right)=-0.433\;J/C$, $\pi^{c}\left(1123.15\;K\right)=-0.742\;J/C$). Furthermore, it can be observed that both the heat flux density and the temperature gradients become larger with increasing electric current density. This can be explained by increasing irreversibilities at higher electric current densities. The case of equal temperatures at the anode and cathode sides of the cell is motivated by the cooling gas within the bipolar plates. Here, the heat transfer on the anode side is much more intense than on the cathode side. If the same heat transfer coefficient is assumed, the temperature at the anode side will be higher. Thus, a new case is studied.

**Figure 6:**
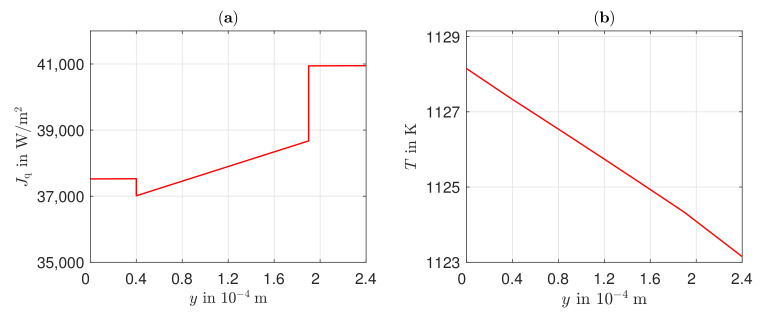
Simulated profile of (**a**) heat flux density and (**b**) temperature along spatial coordinate *y* for a imposed temperature gradient ΔT=5 K between cathode and anode for j=8000 A/$m^{2}$.

In Figure 6, the resulting temperature and heat flux density curves are shown for an imposed temperature difference of 5K between the anode and cathode. An almost linear temperature profile is obtained, wh ich is due to the small temperature differences during normal operation. The heat flux flows continuously from the anode toward the cathode, as the impact of the Peltier effects on the total heat flux decreases, and the heat flux is primarily transported by the temperature gradient. However, the imposed temperature difference of 5K only manifests itself in the course of the temperature and the heat flux density and has no significant influence on the overall operating behavior of the cell.

**Figure 7:**
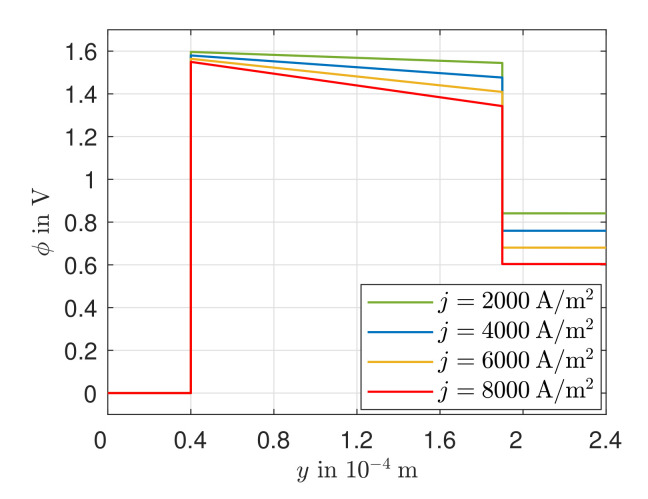
Simulated course of the electric potential field along the spatial coordinate *y*.

Figure 7 shows the simulated electric potential along the spatial coordinate as a function of the electric current density for T(y=0) = $T(y=\Delta y^{a}+\Delta y^{e}+\Delta y^{c})$. In the course of the electrical potential, jumps can be observed at the reaction layers. These result from the equilibrium electrode potentials of the electrodes and the overvoltages that reduce the potential differences at the reaction layers. The electrical potential is greatest in the electrolyte due to the oxygen ion transport that occurs there. With increasing electric current density, greater losses in the electric potential can be observed there, which follow from the irreversible ion transport. The voltage losses due to the charge transport in the GDLs can hardly be observed due to the low electrical resistance. For j=8000 A/$m^{2}$ overvoltages at the electrodes and the ohmic losses across the electrolyte of $\eta^{a}=0.063$ V, $|\eta^{c}|=0.050$ V and $\Delta \phi^{e}=0.2069$ V are obtained. It can be seen that the ohmic losses in the electrolyte are dominant. The fraction of the total voltage losses accounted for by the ohmic losses increases with increasing electric current density, as the activation overvoltages do not increase linearly with increasing electric current density. This dominance of the ohmic losses is due to the limited ionic conductivity of solid oxide ceramics and the strong dependence of the ohmic losses on the electric current density. The overvoltages at the anode, as well as at the cathode, are in the same order of magnitude, but the overvoltage at the anode is slightly greater. This can be explained by the larger exchange current density of the cathode reaction ($j_{0}^{c}=6061.6$ A/m${}^{2}$) as compared to the anode reaction ($j_{0}^{a}=5711.2$ A/m${}^{2}$) at the operating temperature considered here.

### 3.4. Entropy Production Rate

**Figure 8:**
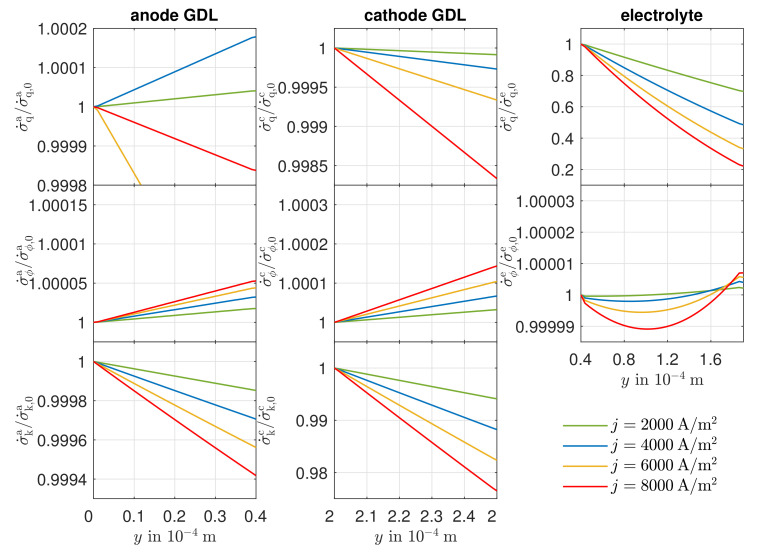
Simulated curves of the local entropy production rate in the anode GDL, the cathode GDL, and the electrolyte along the spatial coordinate *y*.

The local entropy production rates for the anode GDL, the cathode GDL, and the electrolyte are shown in Figure 8 along the spatial coordinate at an operating temperature of 1123.15K. In order to present the curves of the local entropy production rates for different electric current densities in one diagram, the local entropy production rates are each related to their value at the left edge of the respective layer. These absolute values are listed for the electric current densities j=2000 A/$m^{2}$ and j=8000 A/$m^{2}$ in Table 4.

The absolute values of the local entropy production rates of all sections increase in amount with increasing electric current density. The individual contribution of the heat flux density to the entropy production in the cathode GDL are negative and assume larger values in magnitude as the electric current density increases. This is due to the large influence of the Peltier effect, whereby heat is transported toward higher temperatures as a result of charge transport in the GDLs, which leads to negative entropy production rates. The overall entropy production is, of course, always positive. As can already be seen in Figure 5, the Peltier effect has a smaller influence at the anode. With increasing electric current density and, thus, increasing temperature gradient, there is a stronger influence of the Fourier effect. The reduced partial pressure of the oxygen compared to the reactants in the anode GDL leads to a similar magnitude. From Figure 8, it can be seen that, in the anode GDL, the local entropy production rate decreases toward the reaction zone. This fact can be attributed to the increasing partial pressure of the of the water, which causes the local entropy production rate to decrease. Due to the higher total pressure existing in the reaction zone, thus, the local entropy production rate as a result of the water transport predominates. In the cathode GDL, the decreasing partial pressure of oxygen toward the reaction layer leads to increasing local entropy production rates. Despite a constant electrical potential in the GDLs, a change in the local entropy productions due to the electrical current density becomes apparent. The decrease of the absolute temperature leads to an increase of the corresponding force and, thus, to an increase of the local entropy productions. The local entropy productions in the GDLs due to the heat flow can be explained by the different influences of the corresponding forces. In the anode GDL, there is an increase in local entropy production at an electric current density of j=2000 A/$m^{2}$. Here, the increasing corresponding force prevails due to the negatively increasing temperature gradient. In contrast, for j=8000 A/$m^{2}$, the local entropy production decreases due to the predominant decreasing corresponding force due to the increasing absolute temperature. For the electric current densities of j=4000 A/$m^{2}$ and j=6000 A/$m^{2}$, larger changes are seen. For these two operating points, very small temperature gradients and, thus, very small absolute local entropy productions are obtained. In the cathode GDL, the decreasing local entropy production is due to the decreasing magnitude of the heat flux density along the spatial coordinate.

From Table 4, it can be seen that the local entropy production due to the heat flux density is significantly lower than that due to the charge transport. However, with increasing electric current density, a greater deviation from the reference value becomes apparent. At the right edge of the electrolyte, a large part of the heat is transported by the Peltier effect. In the direction of the ion flow, however, the heat to be transported increases due to the irreversibilities, causing the temperature gradient to rise. Since both the flux and its corresponding force increase in the direction of the charge transport, this leads to a strong increase in the local entropy production rate. In the course of the local entropy production rate due to ion transport, a minimum near the left edge of the electrolyte is created. As the electric current density is constant, the cause lies in a variation of the corresponding force. Along the spatial coordinate, the temperature increases monotonically. In contrast, the gradient in the electric potential in the vicinity of the anode side reaction layer indicates smaller potential differences than at the cathode side reaction layer, whereby the increase in absolute temperature initially leads to a decrease in local entropy production, until the corresponding force of the electric potential is predominated by the increase in the amount of the gradient in the electric potential. However, it can be seen from the ordinate that these deviations along the spatial coordinate are quite small.

**Figure 9:**
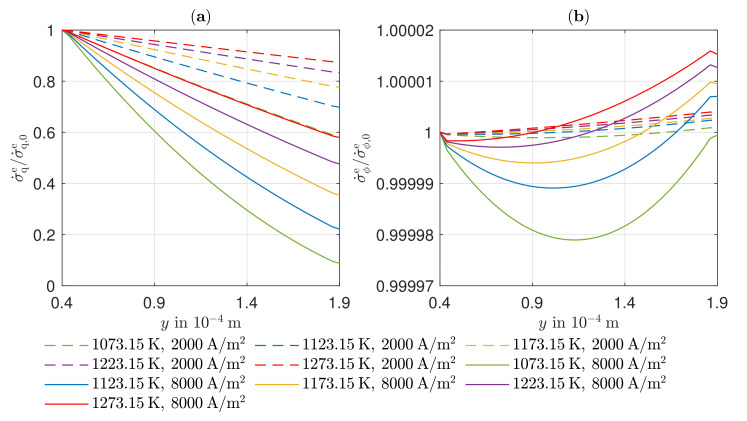
Simulated curve of the local entropy production rate in the electrolyte due to (**a**) heat flux density and (**b**) electrical potential along the spatial coordinate *y* for different current densities *j* and temperatures *T*.

In Figure 9, the curves of entropy production rates in the electrolyte for different operating temperatures at low and high electric current density are shown. The reference values are summarized in Table 5. Increasing operating temperature has a positive effect on the irreversibilities in the electrolyte. In particular, the dominant losses due to ion transport decrease significantly with increasing temperature, which can be explained by the strong temperature dependence of the ionic conductivity of the ceramic electrolyte.

### 3.5. Exergy Analysis

**Figure 10:**
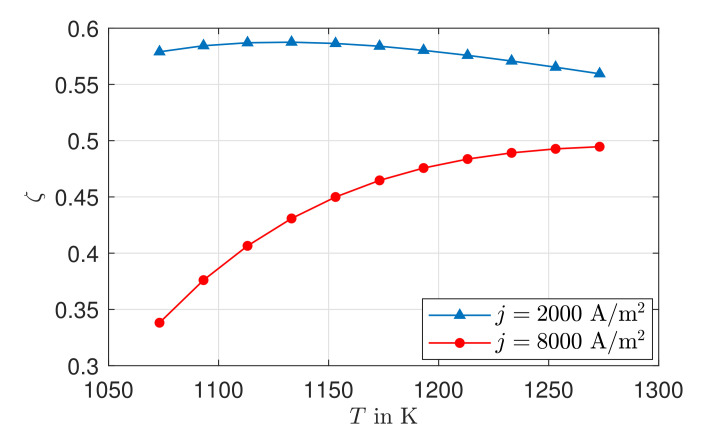
Simulated exergetic efficiency as a function of temperature for j=2000 A/$m^{2}$ and j=8000 A/$m^{2}$.

Figure 10 shows the exergetic efficiencies as a function of operating temperature for a current density of $j=2000\;A/m^{2}$ and $j=8000\;A/m^{2}$. A low electric current density, the exergetic efficiency is initially nearly constant. It decreases with increasing temperature, whereas, at high electric current density, the exergetic efficiency increases monotonically with temperature. This can be explained by two effects. As the operating temperature increases, the ionic conductivity increases, while the dominant losses due to ion transport decrease, resulting in an improvement of the exergetic efficiency. In addition, the physical exergy of the supplied material currents increases with increasing temperature, resulting in a decrease in exergetic efficiency. At low electrical current densities, the increase in the supplied exergy predominates, since the losses due to charge transport are low. This causes the exergetic efficiency to decrease as the operating temperature increases, whereas the positive influence of ionic conductivity predominates at high electric current densities and causes the monotonically increasing curve.

A direct comparison with values from the literature is difficult due to the different assumptions, parameters and operating conditions. Zouhri et al. [53] investigate the exergetic efficiency of a single SOFC cell by varying the parameters for the anode and operating conditions, and they conclude that the porosity and tortuosity of the anode have no effect. With a temperature of T=1273 K, a tortuosity of τ=5, and a porosity of ϵ=0.3, the exergetic efficiency as a function of current density results in $\zeta (j=2000\;A/m^{2})=0.76$ and $\zeta (j=8000\;A/m^{2})=0.61$. Since the same ionic conductivity was assumed in Zouhri et al.’s study as in this study, one possible reason is the thickness of the electrolyte. At 40μm, the thickness of the electrolyte is only one third of the case considered here, which leads to lower losses. This fact is also pointed out by Midilli et al. [54], where the exergetic efficiency for an SOFC stack is investigated under variation of the electrolyte parameter. With a temperature of T=1273 K, an electrolyte thickness of 150μm, and feeding 97% pure hydrogen, the exergetic efficiency is $\zeta (j=2000\;A/m^{2})=0.48$, which, again, is lower than the values gained in this study.

## 4. Conclusions

The aim of this study is to theoretically determine the local entropy production rate of an SOFC single cell at an operating temperature of T=1123.15 K and an operating pressure of p=1 bar. Local entropy production rates can be identified as loss mechanisms, so that this knowledge is of great importance for cell development and optimization of operating strategies. For this purpose, the mass transport in the GDLs of a 1D SOFC model from Reference [12] is described by improved transport equations. The modeling of the GDLs and the electrolyte are based on the NET approach, whereas the reactivity layers are described with the Butler–Volmer approach. The mass transport starting from pure Stefan–Maxwell diffusion is gradually superimposed with further transport mechanisms (Knudsen diffusion, convection, and thermal diffusion) in order to investigate the influences of the individual transport mechanisms. For a comparison of all approaches, the mass transport is additionally modeled with Fick’s law. Furthermore, the transport equations are described as a function of the simulated phenomenological coefficients from Reference [12], with the definitions from Reference [20] as a function of the empirical coefficients. The data for thermal conductivities and ionic conductivity are taken from the literature. The Peltier coefficient is calculated with the approach of Ratkje et al. [14]. For the validation, data for a U,j-characteristic curve of the single cell KeraCell II from the manufacturer Kerafol GmbH and, additionally, self-measured data for the same single cell are used. The calculated curves of temperature, heat flow, electrical potential, and local entropy production rates are discussed. An exergetic analysis is used to assess the efficiency of the cell.

Due to the equimolar composition of the hydrogen and water in the anode, the DGM shows no advantages over the simplified Fick’s law in this particular case. The effect of convection is small compared to the other transport mechanisms because there are low gradients in the total pressure in the anode. There is a higher total pressure in the anode reaction layer than in the gas channel, which is why the gas mixture moves in the direction of the gas channel as a result of convection, thus hindering the mass transport of the hydrogen and improving the mass transport of the water. The influence of Stefan–Maxwell diffusion on the cathode gases is larger, as a significantly smaller effective binary diffusion coefficient results for the cathode-side gas mixture. The improved mass transfer of oxygen by convection is very small in its effect since the gas mixture on the cathode side consists predominantly of nitrogen. On the anode and cathode sides, thermal diffusion has almost no influence on mass transport under the operating conditions considered. The differences between the DGM and the NET approach are negligible in this case.

The existing model is able to represent the U,j-characteristics with a minimum error of 0.08% and a maximum error of 0.93% in a range of the electric current density of j=0–4000 A/$m^{2}$. Due to the negative reaction entropy of the cathode reaction and the irreversibilities, a temperature maximum occurs at the cathode reaction layer. In the cathode GDL, the heat flows toward higher temperatures, which illustrates the significance of the Peltier effects. At the anode reaction layer, net heat is absorbed by the positive reaction entropy. Under an electric current density of j=4000 A/$m^{2}$, Peltier heat absorption outweighs Fourier heat absorption. The electric potential field is significantly influenced by the ohmic losses in the electrolyte. These are always greater than the overvoltages at the electrodes and cause more than half of the voltage losses. This is also shown in the curves of the local entropy production rates. The entropy production rates as a result of the charge transport in the electrolyte are significantly larger in amount than all other losses. The irreversibilities cause 64.44% of the total losses in the cell in the electrolyte at an electric current density of j=8000 A/$m^{2}$ and an operating temperature of T=1123.15. The irreversibilities at the electrodes, together, cause one third of the losses. As the entropy productions in the GDLs are very low, the primary need is to investigate alternative electrolyte materials.

The exergetic efficiency is fundamentally influenced by the operating temperature and the electrical current density. An increase in exergetic efficiency can be achieved through low current densities and higher operating temperatures. 

## Figures and Tables

**Table 1:** General model parameters at T=1123.15.

Parameter	Value	Reference
Dimensions	$\Delta y^{a}=40\;\mu$m	[44]
	$\Delta y^{c}=50\;\mu$m	[44]
	$\Delta y^{e}=150\;\mu$m	[44]
Thermal conductivity	$\lambda^{a}=2\;$ W/m·K	[17]
	$\lambda^{c}=2\;$ W/m·K	[17]
	$\lambda^{e}=1.98\;$ W/m·K	[45]
Entropy of the electrons	$S_{m,e^{-},\mathrm{Pt}}=-1.81$ J/K·mol	[24]
	$S_{m,e^{-},\mathrm{Ni}}=-3.24$ J/K·mol	[25]
Entropy of the ions	$S_{O^{2-}}$ calculated	[14]
Peltier coefficient	$\pi^{a}$ calculated with Equation (23)	
	$\pi^{c}$ calculated with Equation (24)	
	$\pi^{e}$ calculated with Equation (66)	
Ionic conductivity	$\sigma^{e}=2\;$ Wm·K	[17]
Pre-exponential factors	$r^{a,0}=\frac{1}{95\cdot 10^{6}}$ Ω·mK	[22]
for electrical resistances	$r^{c,0}=\frac{1}{42\cdot 10^{6}}$ Ω·mK	[22]
Activation energy	$E_{A,r}^{a}=1150$· R${}_{m}\cdot$ K	[22]
	$E_{A,r}^{c}=1200$· R${}_{m}\cdot$ K	[22]
	$E_{A,j_{0}}^{a}=72,364$ J/mol	[12]
	$E_{A,j_{0}}^{c}=156,306$ J/mol	[12]
Pre-exponential factors	$\gamma^{a}=2,4\cdot 10^{7}$ A/m${}^{2}$	[12]
for exchange current densities	$\gamma^{c}=1.82\cdot 10^{11}$ A/m${}^{2}$	[12]
Penetration coefficients	$\alpha^{a}=0.5$	[15]
	$\alpha^{c}=0.3$	[15]

**Table 2:** Mass transport parameters at T=1123.15 K.

Parameter	Value	Reference
Diffusion volume	$v_{H_{2}}=6.12$	[29]
	$v_{H_{2}O}=13.1$	
	$v_{O_{2}}=16.3$	
	$v_{N_{2}}=18.5$	
Lennard-Jones	$\sigma_{H_{2}}=2.827$ Å, $\epsilon_{H_{2}}/k_{B}=59.7\;K$	[36]
parameter	$\sigma_{H_{2}O}=2.641$ Å, $\epsilon_{H_{2}}/k_{B}=809.1\;K$	
	$\sigma_{O_{2}}=3.467$ Å, $\epsilon_{H_{2}}/k_{B}=106.7\;K$	
	$\sigma_{N_{2}}=3.798$ Å, $\epsilon_{H_{2}}/k_{B}=71.4\;K$	
Pore diameter for GDL	$d_{p}^{i}=5\;\mu$m	[1]
Porosity for GDL	$\epsilon^{i}=0.3$	[1]
	$\tau^{a}=3.15$	[51]
Tortuosity for GDL	$\tau^{c}=2.9$	[52]

**Table 3:** Binary diffusion coefficients ${Đ}_{\mathrm{kj}}^{\mathrm{eff}}$, effective Knudsen diffusion coefficients $D_{k}^{\mathrm{Kn},\mathrm{eff}}$, Fick’s diffusion coefficients $D_{k}^{\mathrm{eff}}$, and thermal diffusion coefficients $D_{k}^{T}$ at T=1123.15 K.

Diffusion coefficients	Value
${Đ}_{H_{2}-H_{2}O}^{\mathrm{eff}}$	0.004 m${}^{2}$/s
${Đ}_{O_{2}-N_{2}}^{\mathrm{eff}}$	$9.78\cdot 10^{-4}$ m${}^{2}$/s
$D_{H_{2}}^{\mathrm{Kn},\mathrm{eff}}$	$5.45\cdot 10^{-4}$ m${}^{2}$/s
$D_{H_{2}O}^{\mathrm{Kn},\mathrm{eff}}$	$1.82\cdot 10^{-4}$ m${}^{2}$/s
$D_{O_{2}}^{\mathrm{Kn},\mathrm{eff}}$	$1.49\cdot 10^{-4}$ m${}^{2}$/s
$D_{N_{2}}^{\mathrm{Kn},\mathrm{eff}}$	$1.59\cdot 10^{-4}$ m${}^{2}$/s
$D_{H_{2}}^{\mathrm{eff}}$	$4.79\cdot 10^{-4}$ m${}^{2}$/s
$D_{H_{2}O}^{\mathrm{eff}}$	$1.74\cdot 10^{-4}$ m${}^{2}$/s
$D_{O_{2}}^{\mathrm{eff}}$	$1.29\cdot 10^{-4}$ m${}^{2}$/s
$D_{N_{2}}^{\mathrm{eff}}$	$1.36\cdot 10^{-4}$ m${}^{2}$/s
$D_{H_{2}}^{T}$	$-2.74\cdot 10^{-7}$ m${}^{2}$/Ks
$D_{H_{2}O}^{T}$	$2.74\cdot 10^{-7}$ m${}^{2}$/Ks
$D_{O_{2}}^{T}$	$6.48\cdot 10^{-9}$ m${}^{2}$/Ks
$D_{N_{2}}^{T}$	$-6.48\cdot 10^{-9}$ m${}^{2}$/Ks

**Table 4:** Reference values of the local entropy production rate at y=0 (anode GDL), $y=0.4\cdot 10^{-4}\;m$ (electrolyte), and $y=1.9\cdot 10^{-4}\;m$ (cathode GDL).

	Anode GDL	Electrolyte	Cathode GDL
	${\dot{\sigma }}_{q,0}^{a}\;\left|\;{\dot{\sigma }}_{\phi ,0}^{a}\;\right|\;{\dot{\sigma }}_{k,0}^{a}$	${\dot{\sigma }}_{q,0}^{e}\;|\;{\dot{\sigma }}_{\phi ,0}^{e}$	${\dot{\sigma }}_{q,0}^{c}\;\left|\;{\dot{\sigma }}_{\phi ,0}^{c}\;\right|\;{\dot{\sigma }}_{k,0}^{c}$
*j* in $A/m^{2}$	in $W/m^{3}K$	in $W/m^{3}K$	in $W/m^{3}K$
2000	−0.04|0.17|4.84	0.18|613.95	−0.15|0.95|4.18
8000	0.64|1.31|77.40	4.40|9822.7	−0.98|17.33|68.15

**Table 5:** Reference values of the local entropy production rate at $y=0.4\cdot 10^{-4}\;m$ (electrolyte) for different operating temperatures at low and high current density.

*j* in $A/m^{2}$	*T* in K	${\dot{\sigma }}_{q,0}^{e}$ in $W/m^{3}K$	${\dot{\sigma }}_{\phi ,0}^{e}$ in $W/m^{3}K$
2000	1073.15	0.19	933.10
2000	1273.15	0.16	233.78
8000	1073.15	5.70	14,928.61
8000	1273.15	3.06	3740.38

## Abbreviations

The following abbreviations are used in this manuscript:

DGM	Dusty-Gas model
GDL	Gas diffusion layer
HOR	Hydrogen oxidation reaction
NET	Non-equilibrium thermodynamics
ORR	Oxygen reduction reaction
PEMFC	Proton exchange membrane fuel cell
SOFC	Solid oxide fuel cell
SOEC	Solid oxide electrolyzer cell
